# Multilevel percutaneous vertebroplasty with the Spine Jack® system in a patient with Cushing disease

**DOI:** 10.1002/ccr3.5034

**Published:** 2021-11-06

**Authors:** Cuauhtemoc Gil‐Ortiz, Alberto Ramírez‐Romero, Dulce Bonifacio‐Delgadillo, Javier Lagos‐Servellon

**Affiliations:** ^1^ Department of Neuroscience Centro Médico Nacional "20 de Noviembre" Mexico City Mexico

**Keywords:** case report, Cushing disease, percutaneous vertebroplasty, Spine Jack system, vertebral compression fractures

## Abstract

**Background:**

Percutaneous vertebroplasty (PV) is a minimally invasive procedure that requires the injection of cement into a fractured vertebral body. Spine Jack® is a vertebroplasty system with an intracorporal implant designed to restore the height of the vertebral body in osteoporotic vertebral fractures. There are no reported cases of PV with Spine Jack® system as treatment for multilevel compression fractures in patients with vertebral osteoporosis due to Cushing disease.

**Case presentation:**

A 55‐year‐old man with lumbago, impaired deambulation 6 weeks prior to presentation, with Oswestry score of 72% and a visual analogue scale (VAS) score of 9 points. Imaging studies showed osteoporotic fractures at T5, T8, T11, T12, and L1‐L5 vertebrae secondary to Cushing disease. PV was performed with a Spine Jack® intracorporal implant device, in three sessions, and multiple levels were operated at each intervention. Post‐operative course demonstrated improvement of pain, height, correction of the kyphotic angle and Oswestry score, without any neurological deficits despite having nine vertebral fractures.

**Conclusion:**

Percutaneous vertebroplasty with the Spine Jack® system is a safe and effective procedure to treat multilevel vertebral fractures due to Cushing disease, improving the quality of life and allowing the patient to remain pain‐free while avoiding major surgery.

## Graphical abstract

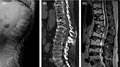

## 1 BACKGROUND

Percutaneous vertebroplasty (PV) is a minimally invasive procedure in which liquid polymethylmethacrylate cement is injected into a fractured vertebral body to relieve pain, reinforce the bone, and prevent further vertebral compression.^1^ Spine Jack® is a vertebroplasty system with an intracorporal implant designed to restore the height of the vertebral body in osteoporotic vertebral fractures, primary or secondary bone tumors, or traumatic fractures.^2^ It is an effective, low‐risk procedure for patients with a significant reduction in pain and analgesic use immediately after surgery that is maintained over time.^3^ Patients treated with Spine Jack® had more efficient height restoration and kyphosis correction and a lower recurrent fracture rate than patients treated with vertebroplasty without increased risk of adjacent or nonadjacent fractures.^4^

In patients with endogenous or exogenous hypercortisolism, bone loss is more severe in trabecular bone than in cortical bone.^5^ Fractures affect about 70% patients with Cushing syndrome. Most of them are vertebral fractures, so patients suffer from back pain and kyphosis together with height loss. The fracture risk is related to the age at onset, disease duration, and severity of the disease, even in cured patients.^6^ The majority of vertebral fractures referred for vertebroplasty are secondary to vertebral insufficiency caused by osteoporosis.^1^ We report the case of a patient with multiple vertebral compression fractures (VCFs) secondary to osteoporosis due to Cushing disease treated with PV with a Spine Jack® system. We did not find case reports of this type in the literature.

## 2 CASE PRESENTATION

**FIGURE 1:**
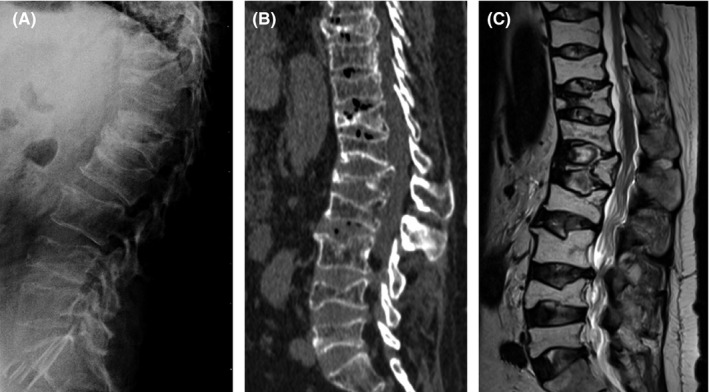
Initial imaging studies. (A) Lateral radiograph shows diffuse osteopenia and biconcave fracture of L1 and L4. (B) Sagittal CT image demonstrates additional compression fractures involving T12, T11, and T8. (C) Sagittal T2‐weighted MRI shows hyperintensity in body of L5, L4, L3, L2, L1, T12, T11, and T8 due to a compression fracture. The patient also had compression fracture at T5 (not shown)

This is a 55‐year‐old man, with central obesity, non‐smoker, auto mechanic technician. In December 2014, he attended the emergency department complaining of serious low back pain without neurological deficit associated with impaired deambulation of 6 weeks secondary to pain. Low back pain started after lifting a heavy object. Five months earlier, he was treated symptomatically for back pain related to domestic activities. On admission, the physical examination demonstrated localized tenderness and percussion pain at lower thoracic and lumbar level, with no restriction of the waist motion. Mild dorsal kyphosis was seen. Muscular tone of the lower limb was normal, and no hypoesthesia nor paresis of the lower limb was observed. There was no evidence of bladder or bowel dysfunction. Physiological reflexes were existent without any pathological ones. We apply the Oswestry score of 72% and a visual analogue scale (VAS) score of 9 points. Initially, plain X‐rays showed a biconcave fracture of L1 with a height loss of 90%, biconcave fracture of L4 and subchondral sclerosis in the endplate of L5 and the superior plates of L3 and L2 (Figure 1A). The computed tomography (CT) scan showed new fractures in T12, T11, T8, and T5 (Figure 1B). Subsequently, we performed a magnetic resonance image (MRI) that confirmed vertebral compression fractures (VCFs) in T5, T8, T11, T12, L1, L2, L3, L4, and L5 (Figure 1C). Patient was elected to undergo surgical repair with ambulatory pre‐surgical work‐up.

**FIGURE 2:**
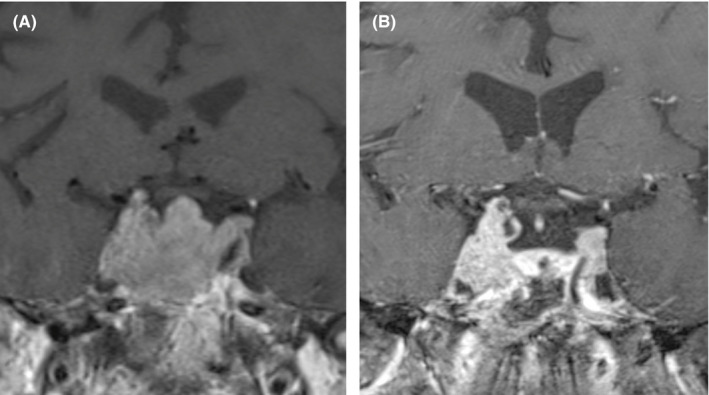
Pre‐ and post‐surgical MRI of the pituitary gland. (A) Post‐contrast coronal T1‐weighted MRI shows sellar mass invading the cavernous sinuses bilaterally. (B) Post‐contrast coronal T1‐weighted MRI after transsphenoidal resection shows persistence of residual tumor in the cavernous sinuses

In the next months, the patient was diagnosed with difficult‐to‐control systemic arterial hypertension. Even though the patient never had exogenous steroid exposure, impaired fasting glucose was found. A bone mineral density measurement was performed which revealed a T‐score of −2.5 in total hip and a T‐score of −3.2 in the left femoral neck, confirming suspicion of osteoporosis. His base morning cortisol and adrenocorticotropic hormone (ACTH) were found to be 1,625 (normal range 126–662) nmol/L and 62.2 (normal range 0–35) pmol/L, respectively. Cortisol was suppressed to 86% with high‐dose dexamethasone. A brain MRI was performed that showed a mass in the sellar region measuring 29 mm with invasion of both cavernous sinuses, suggestive of pituitary macroadenoma (Figure 2A). Finally, we concluded that it was Cushing disease caused by an ACTH‐secreting pituitary adenoma. Multiple osteoporotic VCFs were found to be secondary to this disease.

**FIGURE 3:**
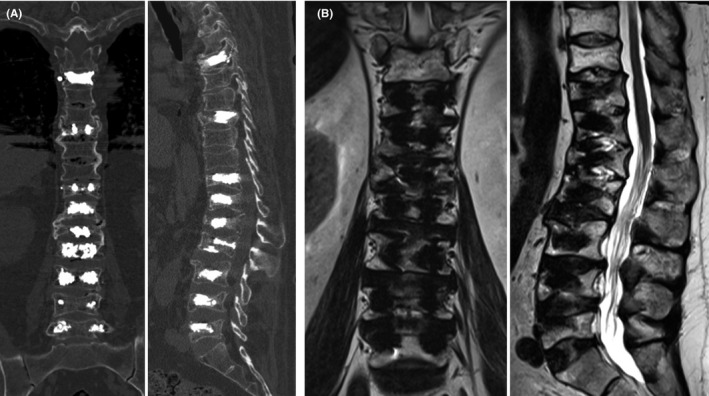
Imaging studies after the Spine‐Jack® procedure. (A, B) CT and T2‐weighted MRI show post‐procedure changes in the bodies of T5, T8, T11, T12, L1, L2, L3, L4, and L5, which correspond to the Spine‐Jack® system

With diagnosis of nine VCFs, we decided PV as treatment which was performed with a Spine Jack® intracorporal implant device. In May 2016, a 3‐step staged repair was decided at multiple levels, as follows: (a) T8, T11, L1, and L4; volumes of cement used were 4, 4, 4, and 3 ml, respectively; (b) L2, L3, and L5; volumes of cement used were 3, 4, and 4 ml, respectively; and (c) T5 and T12; volumes of cement used were 3.8 and 4 ml, respectively. The patient had minor leakage of cement from T5 without motor weakness, and he was treated with a laminectomy and remotion of the leaked cement. Spine CT and leaked cement. Spine CT and MRI (Figure 3A,B) were performed after the last Spine‐Jack® procedure.

**FIGURE 4:**
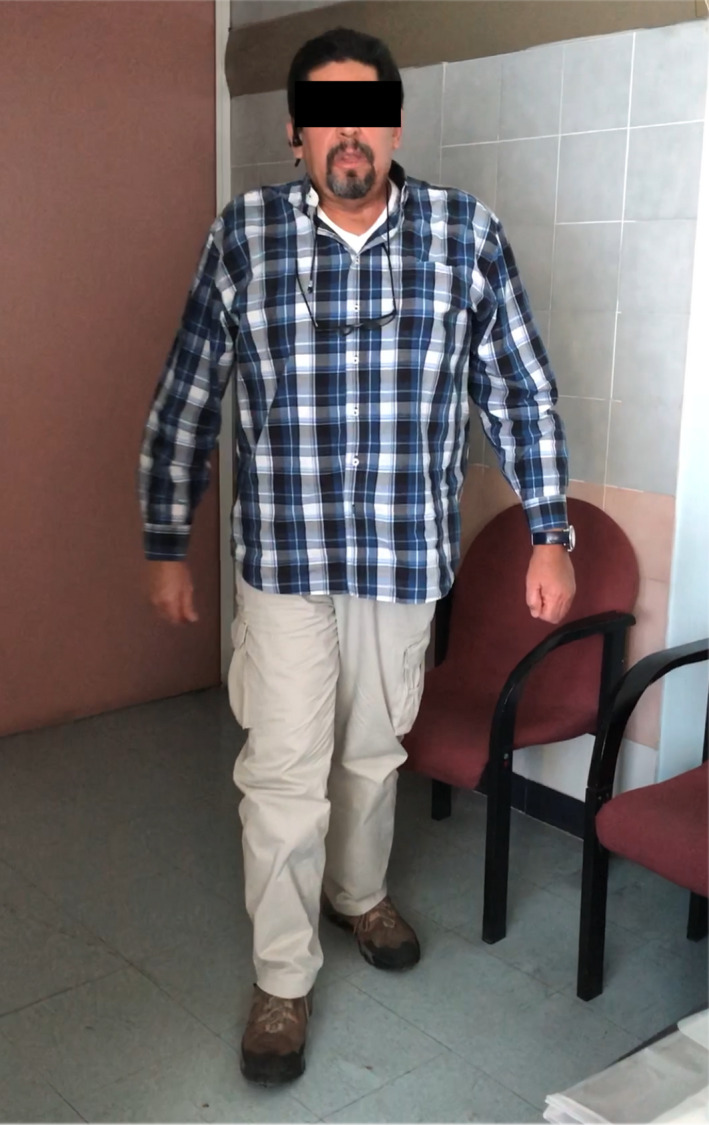
Patient showed a 30‐point improvement for Oswestry score, VAS score of 0, and achieved autonomous ambulation after PV of nine VCFs

The post‐operative outcome after the treatment of nine chronic VCFs showed an improvement of 30 points for the Oswestry score, a VAS score of 0 and the patient achieved autonomous ambulation (Figure 4). A partial restoration of vertebral height with craniocaudal expansion was obtained holding the axial vector. At 33 months of follow‐up, an increase of the mid vertebral height at thoracic levels of 4.75 mm on average was documented (Table 1). After four years, we found no decrease in vertebral height, an improvement of 7° in the kyphotic angle, and absence of new fractures. In December 2016, the patient underwent transsphenoidal resection of the sellar lesion and pathology was adenoma. Post‐surgery MRI shows persistence of residual tumor in the cavernous sinuses (Figure 2B). The patient has not yet achieved the cure criteria for Cushing disease.

**TABLE 1:** Increase in vertebral height in millimeters post‐vertebroplasty at 33 months of follow‐up.

Level	Anterior vertebral height	Mid vertebral height	Posterior vertebral height
T5	3	8	2
T8	1	9	3
T11	1	1	2
T12	4	1	0
L1	1	6	2
L2	1	4	1
L3	1	4	0
L4	1	5	10
L5	0	2	2
Thoracic average	2.25	4.75	1.75
Lumbar average	0.8	4.2	3

## 3 DISCUSSION

One of the first symptoms in patients with Cushing disease is the fracture of long bones or vertebrae. Therefore, it is common to find fractures before the diagnosis, so it is necessary to perform an exhaustive search for underlying causes in patients who arrive with multiple nontraumatic fractures. Our patient had limitations in mobility, collapse of the vertebral body resulting in a kyphotic deformity, and loss of autonomy. It was difficult to identify the location of the fracture because patients with multiple fractures do not present with the typical clinical presentation. After PV, the patient had partial correction of kyphosis and remains without neurological deficits despite having nine chronic VCFs. Inherent to the treatment, there is a risk of new fractures, mainly in patients with multilevel treatment. However, after four years of VP, the patient had not presented with new vertebral fractures.

Patients treated with PV had statistically significant improvements in pain relief and a similar incidence of adjacent vertebral fracture compared with patients who underwent traditional treatment.^7^ The time of fracture is important for pain relief; the indication for treatment must focus on the fracture age: acute (6 weeks), subacute (6–12 weeks), and chronic (12 weeks). Evidence has shown that patients with severe pain treated within the first 6 weeks of fracture are suitable to undergo PV.^8^ The VERTOS,^9^ FREE,^10^ INVEST,^11^ and KAVIAR^12^ studies showed a good outcome in patients treated with PV compared with the outcomes for patients who underwent different treatments for osteoporotic VCFs. A meta‐analysis comparing vertebroplasty and kyphoplasty did not show any differences in back pain or the disability pain scores at any time point^13^; kyphoplasty is superior to vertebroplasty in restoring the height of vertebrae (88–93%), but the control of pain is similar for both (90–95%).^14^ PV may be the best way to relieve pain; conservative treatment might lead to decrease the incidence of new fractures, and balloon kyphoplasty might have the lowest risk of all‐cause discontinuation in older people with osteoporotic VCFs.^15^ Some studies have shown that the endplate fracture reduction gained by inflation one tamps cannot be maintained after deflation.^16^, ^17^

During VP, the high‐pressure injection of low viscosity cement directly into the cancellous bone makes it difficult to control the cement in the vertebral body. The risk of cement leaking outside the vertebral body is unpredictable.^18^ According to recent results, the rates of cement leaking may reach 65%.^19^ Major complications can be cement embolism as Rahimi reported a case in 2018.^20^

Rashid presented similar case of 36‐year‐old women with VCFs secondary to hypercortisolism induced by a bronchial carcinoid tumor.^21^ Furthermore, Tian reported a case of multilevel VCFs related to chronic glucocorticosteriod use.^22^ In none of these cases did they achieve PV with the Spine Jack® system. The maximum number of vertebrae that can be injected in one session is debatable; single‐level injection is associated with better outcomes than multilevel injection.^23^ Some studies have suggested doing no more than three levels of injection during one session to reduce the complications associated with PV and to avoid patient discomfort.^24^ Zoarski suggested that only five levels can be treated simultaneously and that the use of eight levels is not acceptable.^25^ Mailli found no difference in PV with more than three levels per session.^26^ A meta‐analysis suggested that the intravertebral cleft, cortical disruption, low cement viscosity, and high volume of injected cement may constitute a high risk for cement leak after vertebroplasty or kyphoplasty. The patient's age, sex, and fracture type, as well as the operation level and surgical approach, were not significant risk factors.^27^

To prevent new fractures, prophylactic PV to adjacent vertebrae is recommended.^28^ Prediction of which vertebrae are at risk is difficult, and prophylactic vertebroplasty does not avoid the risk of recurrence.^29^ The incidence of new vertebral fractures adjacent or distant to the fractured one after PV ranges between 7% and 37%.^30^ It is still unclear whether new fractures are related to the natural history of the underlying disease or to the treatment.^24^ The incidence of new fractures after PV varies between 7.8%^26^ and 37%.^31^

## 4 CONCLUSION

Based on this case, we suggest that PV with the Spine Jack® system is a safe and effective treatment option for multilevel VCFs due to Cushing disease; however, more cases are needed to be treated in order generalize this type of treatment. Minimal invasion is an excellent option to treat patients with underlying diseases that cause fractures such as Cushing disease, thus avoiding major surgery. This report allowed us to achieve four aims, as follows: pain management, height vertebral increase, correction of the kyphotic angle, and improvement of the Oswestry score.

## CONFLICT OF INTEREST

None.

## AUTHOR CONTRIBUTIONS

CGO made contribution in the conception of the study. DBD, CGO, ARR, and JLS made contribution in the collection and analysis of data. All the authors made contribution on the drafting of the manuscript. DBD and CGO critically revised the manuscript. All the authors agreed in the final version to be published and agreed be accountable for all aspects of the work in ensuring that questions related to the accuracy or integrity of any part of the work.

## ETHICAL APPROVAL

The authors declare that the data collected, analysis, and disclosure were made under the ethics protocols and under the supervision of the ethics committee of Centro Medico Nacional 20 de noviembre; governed by the Declaration of Helsinki.

## CONSENT

The study participant signed with full knowledge an informed consent on the clinical and imaging data before publication, fully preserving the patient's anonymity. The authors confirm the patient consent has been signed and collected in accordance with the journal's patient consent policy.
